# Supplementary material for: Effect of montelukast in preventing dengue with warning signs among patients with dengue: A multicenter, randomized, double-blind, placebo-controlled trial
Source: PLoS Negl Trop Dis. 2024 Feb 2;18(2):e0011927. doi: 10.1371/journal.pntd.0011927 (PMC10866515; doi:10.1371/journal.pntd.0011927)
Supplement: S1 Protocol — (PDF) [file pntd.0011927.s002.pdf]

Study Protocol

Study title

Effect of montelukast in preventing dengue with warning signs in dengue patients: a randomized, double-blind, placebo controlled, superiority trial

Study Investigators

Chief Investigator:

LTC Vasin Vasikasin, MD
Infectious Diseases Division, Phramongkutklao Hospital
Contribution: Study design, study supervision, protocol preparation, IRB application, study registration, research grant application, research grant administration, central data review and analysis

Principal Investigators (PI) and Sub-Investigator (SI):

Phramongkutklao Hospital

- Principal Investigator: MAJ Worapong Nasomsong, MD
- Sub-Investigator: Nattapat Nitinai, MD

Ananda Mahidol Hospital

- Principal Investigator: MAJ Worayon Chuerboonchai, MD
- Sub-Investigators: LT Vutthikorn Khingmontri, MD, LT Bawornnan Panuvatvanich, MD

Fort Suranari Hospital

- Principal Investigator: COL Thananut Bangchuad, MD
- Sub-Investigators: LT Tanapol Roongfa-ngam, MD, Menanchaya Pongpricharoen, MD

Fort Hatyai Hospital

- Principal Investigator: Sorawat Sangkaew, MD PhD

Contribution of PI: Study oversight, analysis of data
Contribution of SI: Data collection

Study Coordination Centers

For general queries, supply of trial documentation, and collection of data, please contact:
LTC Vasin Vasikasin
Infectious Diseases Division, Phramongkutklao Hospital; Bangkok, Thailand, 10400

Funder

Royal College of Physicians of Thailand and Phramongkutklao College of Medicine

Number of sites: 4
Phramongkutklao Hospital; Bangkok, Thailand, 10400
Ananda Mahidol Hospital; Lopburi, Thailand, 15000
Fort Suranari Hospital; Nakhon Ratchasima, Thailand, 30000
These hospitals are subject to superintendence of Army Medical Department, Royal Thai Army, Thailand
Hatyai Hospital; Songkhla, Thailand, 90110, under the Ministry of Public Health, Thailand

Institutional Review Board, Royal Thai Army Medical Department identification number: S077h/63

ClinicalTrials.gov identification number: NCT04673422

Study duration and dates

The study will start July 1st, 2020. Patient recruitment are proposed to last 25 months to start in January 2021 and end in January 2023. Data analyses, data cleaning, analysis and reporting will take a further 3 months.

Introduction and Study Rationale

Dengue has been the growing public health problem in many tropical countries, including Thailand. (1) Almost 4 billion people were estimated to be at risk, with estimated 400 (95%CI: 284-528) million infections occurring annually. In Asia, around 10% of febrile patients were virologically confirmed with dengue. (2) In Thailand, Department of Disease Control reported the annual incidence between the year of 2015 and 2019 was 20 people per 100,000 population, with 10-20 annual mortality.

The most common cause of death is from dengue shock as a result of vascular leak syndrome. (3) This condition can occur in various clinical manifestations ranging from mild cases to life-threatening condition of dengue shock syndrome. The common sites of plasma leakage are pleural effusion and ascites (4) The contributing factors for endothelial dysfunction in dengue are cytokines such as soluble tumor necrosis factor receptor (sTNFR/75), interferon gamma, and vascular endothelial growth factor (4-7), NS1 antigenemia, complement activation, (8, 9) and activation of dendritic cells, macrophages, and mast cells (10)

Mast cells have recently been acknowledged as an important regulator for promoting innate immune responses. (11) Important composition of granules in mast cells are proteases, chymase and tryptase, histamine, heparin (12, 13) and leukotriene. (14) The activated mast cells can undergo degranulation, releasing these cytokines. These increase capillary permeability, leading to vascular leakage. (14, 15)

Leukotriene has an important role in promoting plasma leakage and leukocyte adhesion in postcapillary venules. (16) In dengue patients, leukotriene levels usually elevate during febrile and defervescence stage for 35 and 38 times of the baseline values, and return to baseline in convalescence stage. (17) Blocking leukotriene in dengue infected mice can significantly reduce plasma leakage (14).

The management of dengue consists of only symptomatic treatment, and intravenous fluid replacement. No specific treatment has yet been demonstrated of a benefit in preventing complications. (3) In the recent decades, mast cells have been demonstrated as a major contributor of severe forms of dengue, (10, 18) leading to research in reduction of vascular permeability with mast cell stabilizers or anti-histamine drugs. An animal model studies found that a tryptase inhibitor, nafamostat, (19) or leukotriene inhibitor, montelukast, could reduce the plasma leakage. (14)

In 2018, an open-label study found that patients with montelukast had a 22% absolute risk reduction in dengue shock syndrome, compared to standard treatment. (20) However, there has never been any randomized controlled trial evaluating the efficacy of montelukast in dengue patients.

Study Design and Procedures

This randomized, prospective, 2-arm, parallel-group, double-blind, placebo-controlled superiority trial will evaluate the efficacy of montelukast in decreasing disease severity of dengue patients. The primary objective is to determine whether montelukast is more effective than placebo in reducing dengue with warning signs. Other important study objectives are to access the efficacy of montelukast in decreasing hemoconcentration, thrombocytopenia, hospitalization, length of hospital stay, severe dengue, dengue shock, and mortality.

The study cohort will include 358 patients aged 20 years or above with positive NS1 antigen test. Participants will be recruited over a 2-year period at 2 hospitals and will be followed up for up to 14 days.

The objectives are as follows:

Primary Hypothesis

In dengue patients aged 20 years or above with positive NS1 antigen, does montelukast reduce the incidence of dengue warning signs? This hypothesis will be tested using a composite outcome including

- abdominal tenderness or pain
- persistent vomiting
- clinical fluid accumulation
- mucosal bleeding
- liver enlargement >2cm
- increase in hematocrit concurrent with decrease in platelet count

However, lethargy will be excluded as a criterion for warning sign as almost all patients reported subjective lethargy. (21)

Secondary Hypotheses

The secondary hypotheses will address secondary outcome in analyses designed to support and confirm the primary analysis. These will include components of composite outcomes of dengue with warning signs, hospitalization, length of hospital stay, severe dengue, dengue shock, and mortality.

Eligibility Criteria

The objective of setting inclusion/exclusion criteria is to identify a trial population that will ensure adequate event rates for statistical power and maximize safety. Inclusion/exclusion criteria were made as simple as possible to ensure standard implementation across all study sites. Specifically, the eligibility criteria were developed to identify patients with confirmed early dengue infection, hence, the trial population is comprised of individuals with NS1 antigenemia, which is usually found in a few days after symptom onset.

Inclusion Criteria

- at least 20 years old
- diagnosis of dengue
- positive NS1 or PCR test

Exclusion Criteria

- any warning sign of dengue
- concurrent diagnosis of other causes of fever, such as malaria or heat stroke
- pregnancy
- being unable to take medication by mouth
- critical illness needing intubation or admission to an intensive care unit
- being unable to communicate
- other indication of montelukast

Recruitment: Informed Consent, Screening, Baseline

Recruitment

The goal of participant recruitment is to create a trial population that will ensure adequate event rates for statistical power while maximizing participant safety to the population for which the intervention is intended. Recruitment strategies may also include dissemination of information about this trial to laboratory professionals who test NS1 antigen from the patient specimens.

Regulatory and Ethical Considerations, including the Informed Consent Process

The study will be conducted in accordance with Good Clinical Practice (GCP), all applicable subject privacy requirements, and the guiding principles of Helsinki, including Institute Review Board (IRB) of both the Royal Thai Army Medical Department and related hospitals review and approval of study protocol and any subsequent amendments.

Existing Populations in the Clinical Site Practices

Methods for identifying potentially eligible participants within the clinical practice of the research settings is a targeted alert of positive laboratory results by attending physicians.

Baseline Information

Once local regulatory requirements have been approved, investigator plans to identify potential study patients may be implemented. Participant informed consent must also be obtained prior to performing any procedures related to the trial. The following key elements are outlined in the study assessments and procedures below:

1. Verify participant's interest in study.
2. Obtain study consent
3. Continue collection of screening information, including such items as contact information, additional eligibility information, and medical history.
4. Confirmation that all inclusion/exclusion criteria satisfied
5. Completion of the study randomization procedure and baseline data collection

The following information will be recorded on the case record form by the attending clinicians:

- Patient details (e.g. date of birth, sex, weight)
- Dengue symptom onset date
- Major comorbidity (e.g. heart disease, diabetes, chronic lung disease)
- Date of hospitalisation
- Laboratory results
- Dengue serotypes
- Name of person completing the form

Randomization and allocation concealment

Eligible patients will be randomized using a computer-generated randomisation list prepared by an independent statistician and stratified by the centre with a block size of four in a 1:1 ratio to one of the two treatment arms:

Arm 1: montelukast 10 mg

Arm 2: placebo 10 mg

Administration of allocated treatment

Study medication will be given 10 mg orally immediately and every day thereafter for 10 days or until recovery, defined as the discontinuation of the follow up appointment by the attending physicians, whichever is shorter.

Collecting follow-up information

The following information will be ascertained at least every other day for 14 days or until the discontinuation of the follow up appointment by the attending physicians, whichever is shorter.

- Clinical symptoms and signs of warning signs

- Hospitalisation status, death status
- Laboratory results

Follow-up information is to be collected on all study participants, irrespective of whether or not they complete the scheduled course of allocated study treatment. Study staff will seek follow-up information through various means including medical staff, reviewing information from medical notes, routine healthcare systems, and registries.

Duration of follow-up

All randomised participants are to be followed up until the discontinuation of the follow up appointment by the attending physicians, whichever is shorter. It is recognised that in the setting of this trial, there may be some variability in exactly how many days post-randomisation information on disease status is collected. This is acceptable and will be taken account of in the analyses and interpretation of results, the principle being that some information about post-randomisation disease status is better than none.

Withdrawal of consent

A decision by a participant that they no longer wish to continue receiving study treatment should not be considered to be a withdrawal of consent for follow-up. However, participants are free to withdraw consent for some or all aspects of the study at any time if they wish to do so. In accordance with regulatory guidance, de-identified data that have already been collected and incorporated in the study database will continue to be used (and any identifiable data will be destroyed).

Patients should be withdrawn from study treatment if the following complications occur:

- Development of behavioural change, hallucinations, depression, suicidal thoughts and actions, sleepwalking, stuttering, tremor or shakiness, trouble sleeping, uncontrolled muscle movements
- Potential accidental administration of a dose of >10mg of montelukast a day (due to error in administration of study medication or accidental administration of non-study montelukast)

Figure 1 summarises the study design, Table 1 summarises the procedure.

Figure 1: study design

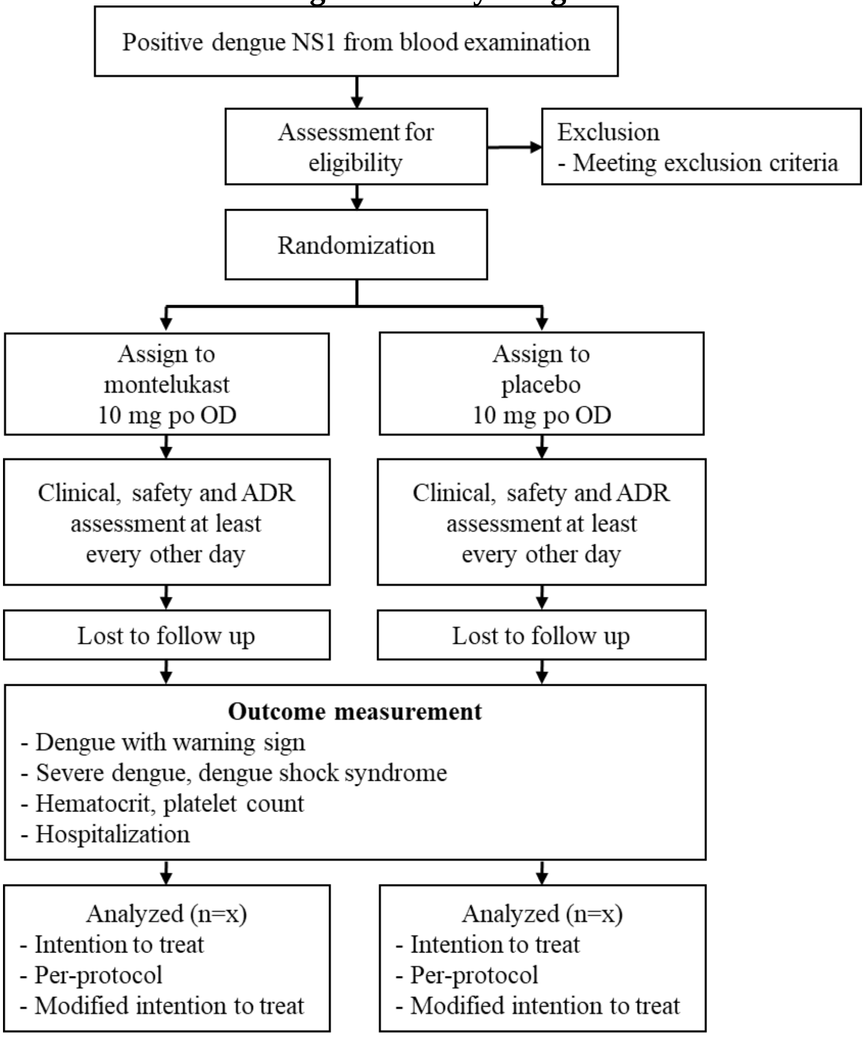

Table 1: Trial procedure

**Procedure**	D1	D2-13	D14
Informed consent	x		
Review of eligibility criteria	x		
Randomization	x		
Demography, medical history, comorbidities	x		
Physical examination	x	x*	
Clinical evaluation	x	x*	
Pregnancy test in women	x		
CBC	x	x*	
dengue RT-PCR	x		
Side effects monitoring	x	x*	x
Warning signs, severe dengue, and dengue shock		x*	x
Admission status		x*	x

*outpatients visit at least every other day, or daily inpatient visit until recovery

Statistical Analysis

Sample size and power

Based on findings in a study reporting the incidence of dengue with warning signs excluding lethargy, (21) we have assumed a 54.5% event rate of the primary outcome in the standard group. Regarding the 22% absolute risk reduction in dengue shock syndrome in an open-label study, (20) we have assumed a 15% effect size for the intervention (absolute risk reduction of 0.15). With a 5% rate of loss to follow-up, and a one sided test at the 2.5% level, a sample size of 358 provides approximately 80% power to detect a reduction in the primary endpoint between the two arms.

Outcomes

The **primary objective** is to provide reliable estimates of the effect of montelukast on the incidence of dengue warning signs, which will be assessed during the defined period, 14 days or until the discontinuation of the follow up appointment by the attending physicians, whichever is shorter. Those who missed the follow-up appointment will be recorded as missing values after the time of the last known status.

The **secondary objectives** are to assess the effects of montelukast on components of composite outcomes of dengue with warning signs, hospitalization, length of hospital stay, severe dengue, dengue shock, and mortality.

Methods of analysis

Comparisons will be made between all participants randomized to the different treatment arms, irrespective of whether they received their allocated treatment ("intention-to-treat" analyses).

The primary analysis will apply Cox proportional hazards regression to all randomized participants to compare the time from randomization to the first occurrence of dengue with warning signs between the two treatment groups. The model will include an indicator for intervention arm as its sole predictor variable. Clinical site at randomization will be a stratifying factor. Follow-up time will be censored at the last date of event ascertainment. The p-value from the primary analysis will be based on the chisquare statistic from a likelihood ratio test obtained from proportional hazards models with and without the term for intervention arm. This likelihood ratio test will constitute the primary test of statistical significance for the primary analysis. Tests of heterogeneity or trend will generally be used to assess disparity in efficacy among different subgroups (e.g. men vs. women; age group, high vs. low viral load, dengue serotypes). One sided tests at the 0.025 level are used.

The secondary analysis will be analyzed using chi-square test or Fisher exact test for categorical variables, and Student T-test or Wilcoxon rank-sum test for continuous variables. These will be reported with 95% confidence intervals and nominal p-values.

Data and safety monitoring board

An independent Data and Safety Monitoring Board will be established to monitor data and oversee participant safety. Members will be appointed by the Research Unit, Phramongkutklao Hospital to provide oversight of the trial. Meetings of the DSMB will be held at least every 12 months. Material for these meetings will be distributed two weeks in advance of the meetings. Up-to-date statistical analyses will be provided to the DSMB in preparation for their meetings. The analyses will include data on recruitment, outcome measures, any side-effects or safety concerns, and will be designed in cooperation with the DSMB. Interim analyses of the intervention effectiveness will be performed at times coinciding with the meetings of the DSMB, and will be controlled to protect the overall Type

I error of the trial. The planned interim analysis is at 50% and 75% of the enrollment, with alpha spending by Lan-DeMets spending function with O'Brien-Fleming type boundaries (α=0.006, α=0.018, and α=0.026, respectively)

These results will be for the use of the DSMB and will not be revealed to the investigators. The purpose of these analyses will be for the DSMB to assess the trial progress with respect to intervention efficacy and safety, for possible recommendations regarding early termination of the trial.

Ethical Aspects

Ethical principles

This study will be conducted in accordance with the principles laid down by Thai Good Clinical Practice Guidelines and other Local Ethics Committees.

Consent

The research coordinator will approach eligible patients as soon as their blood are positive for NS1 to obtain informed consent. Patients must be enrolled within 24 hours of positive NS1 antigen. Critically ill patients are excluded.

Expected adverse events

Montelukast is well tolerated in in patients with asthma. (22) There were no significant differences between the montelukast and placebo treatment groups in the frequency of any adverse effect, with the exception of allergic rhinitis, which occurred more frequently in placebo than montelukast recipients.

In 2020, FDA required Boxed Warning about serious mental health side effects for montelukast, after 82 cases identified of completed suicide associated with montelukast, with many reporting the development of concomitant neuropsychiatric symptoms prior to the event. (23) Patients were suggested to stop taking montelukast and notify a health care professional if they experience behavior or mood-related changes while taking the medicine.

At each visit, the trial staff will specifically query participants for serious adverse events (SAEs). In addition, information on serious adverse events may also be reported to study staff spontaneously by participants through telephone calls. SAEs will be collected and reported from screening to the end of the study follow-up period for an individual participant. SAEs will be followed until resolution, stabilization, or until it is determined that study participation is not the cause. Reports of serious adverse events will be collected for review by the DSMB at their meetings.

References

1. Guzman MG, Gubler DJ, Izquierdo A, Martinez E, Halstead SB. Dengue infection. Nat Rev Dis Primers. 2016;2:16055.
2. L'Azou M, Moureau A, Sarti E, Nealon J, Zambrano B, Wartel TA, et al. Symptomatic Dengue in Children in 10 Asian and Latin American Countries. N Engl J Med. 2016;374(12):1155-66.
3. Wilder-Smith A, Ooi EE, Horstick O, Wills B. Dengue. Lancet. 2019;393(10169):350-63.
4. Srikiatkhachorn A, Krautrachue A, Ratanaprakarn W, Wongtapradit L, Nithipanya N, Kalayanarooj S, et al. Natural history of plasma leakage in dengue hemorrhagic fever: a serial ultrasonographic study. Pediatr Infect Dis J. 2007;26(4):283-90; discussion 91-2.
5. Bethell DB, Flobbe K, Cao XT, Day NP, Pham TP, Buurman WA, et al. Pathophysiologic and prognostic role of cytokines in dengue hemorrhagic fever. J Infect Dis. 1998;177(3):778-82.
6. Srikiatkhachorn A, Green S. Markers of dengue disease severity. Curr Top Microbiol Immunol. 2010;338:67-82.
7. Green S, Vaughn DW, Kalayanarooj S, Nimmannitya S, Suntayakorn S, Nisalak A, et al. Early immune activation in acute dengue illness is related to development of plasma leakage and disease severity. J Infect Dis. 1999;179(4):755-62.
8. Avirutnan P, Punyadee N, Noisakran S, Komoltri C, Thiemmeca S, Auethavornanan K, et al. Vascular leakage in severe dengue virus infections: a potential role for the nonstructural viral protein NS1 and complement. J Infect Dis. 2006;193(8):1078-88.
9. Nascimento EJ, Silva AM, Cordeiro MT, Brito CA, Gil LH, Braga-Neto U, et al. Alternative complement pathway deregulation is correlated with dengue severity. PLoS One. 2009;4(8):e6782.
10. Londono-Renteria B, Marinez-Angarita JC, Troupin A, Colpitts TM. Role of Mast Cells in Dengue Virus Pathogenesis. DNA Cell Biol. 2017;36(6):423-7.
11. St John AL, Rathore AP, Yap H, Ng ML, Metcalfe DD, Vasudevan SG, et al. Immune surveillance by mast cells during dengue infection promotes natural killer (NK) and NKT-cell recruitment and viral clearance. Proc Natl Acad Sci U S A. 2011;108(22):9190-5.
12. Schmutzler W, Bolsmann K, Zwadlo-Klarwasser G. Comparison of histamine release from human blood monocytes, lymphocytes, adenoidal and skin mast cells. Int Arch Allergy Immunol. 1995;107(1-3):194-6.
13. Marone G, Varricchi G, Loffredo S, Granata F. Mast cells and basophils in inflammatory and tumor angiogenesis and lymphangiogenesis. Eur J Pharmacol. 2016;778:146-51.
14. St John AL, Rathore AP, Raghavan B, Ng ML, Abraham SN. Contributions of mast cells and vasoactive products, leukotrienes and chymase, to dengue virus-induced vascular leakage. Elife. 2013;2:e00481.
15. Syenina A, Jagaraj CJ, Aman SA, Sridharan A, St John AL. Dengue vascular leakage is augmented by mast cell degranulation mediated by immunoglobulin Fcgamma receptors. Elife. 2015;4.
16. Dahlen SE, Bjork J, Hedqvist P, Arfors KE, Hammarstrom S, Lindgren JA, et al. Leukotrienes promote plasma leakage and leukocyte adhesion in postcapillary venules: in vivo effects with relevance to the acute inflammatory response. Proc Natl Acad Sci U S A. 1981;78(6):3887-91.
17. Loke WM, Chow AY, Lam Mok Sing K, Lee CY, Halliwell B, Lim EC, et al. Augmentation of 5-lipoxygenase activity and expression during dengue serotype-2 infection. Virol J. 2013;10:322.

18. Sherif NA, Zayan AH, Elkady AH, Ghozy S, Ahmed AR, Omran ES, et al. Mast cell mediators in relation to dengue severity: A systematic review and meta-analysis. Rev Med Virol. 2020;30(1):e2084.

19. Rathore AP, Mantri CK, Aman SA, Syenina A, Ooi J, Jagaraj CJ, et al. Dengue virus-elicited tryptase induces endothelial permeability and shock. J Clin Invest. 2019;129(10):4180-93.

20. Ahmad A, Waseem T, Butt N, Randhawa F, Malik U, Shakoori T. Montelukast Reduces the Risk of Dengue Shock Syndrome in Dengue Patients. Tropical biomedicine. 2019;35:1115-22.

21. Leo YS, Gan VC, Ng EL, Hao Y, Ng LC, Pok KY, et al. Utility of warning signs in guiding admission and predicting severe disease in adult dengue. BMC Infect Dis. 2013;13:498.

22. Price D. Tolerability of montelukast. Drugs. 2000;59 Suppl 1:35-42; discussion 3-5.

23. USFDA. Boxed Warning about serious mental health side effects for asthma and allergy drug montelukast (Singulair); advises restricting use for allergic rhinitis, March 3, 2020. Available from: https://www.fda.gov/media/135840/download.
