# Supplementary figures and images for: Effect of montelukast in preventing dengue with warning signs among patients with dengue: A multicenter, randomized, double-blind, placebo-controlled trial
Source: PLoS Negl Trop Dis. 2024 Feb 2;18(2):e0011927. doi: 10.1371/journal.pntd.0011927 (PMC10866515; doi:10.1371/journal.pntd.0011927)

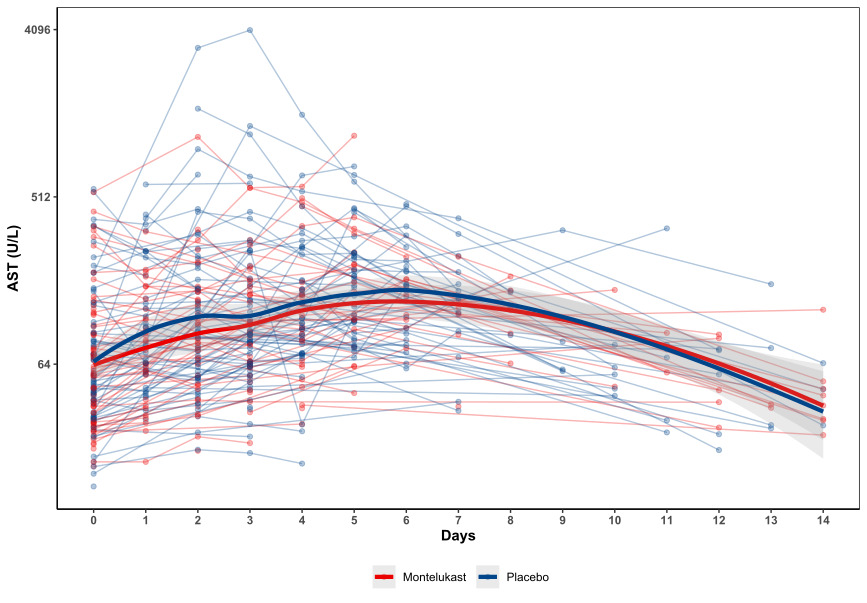

Supplement: S1 Fig — (JPG) [file pntd.0011927.s003.jpg]

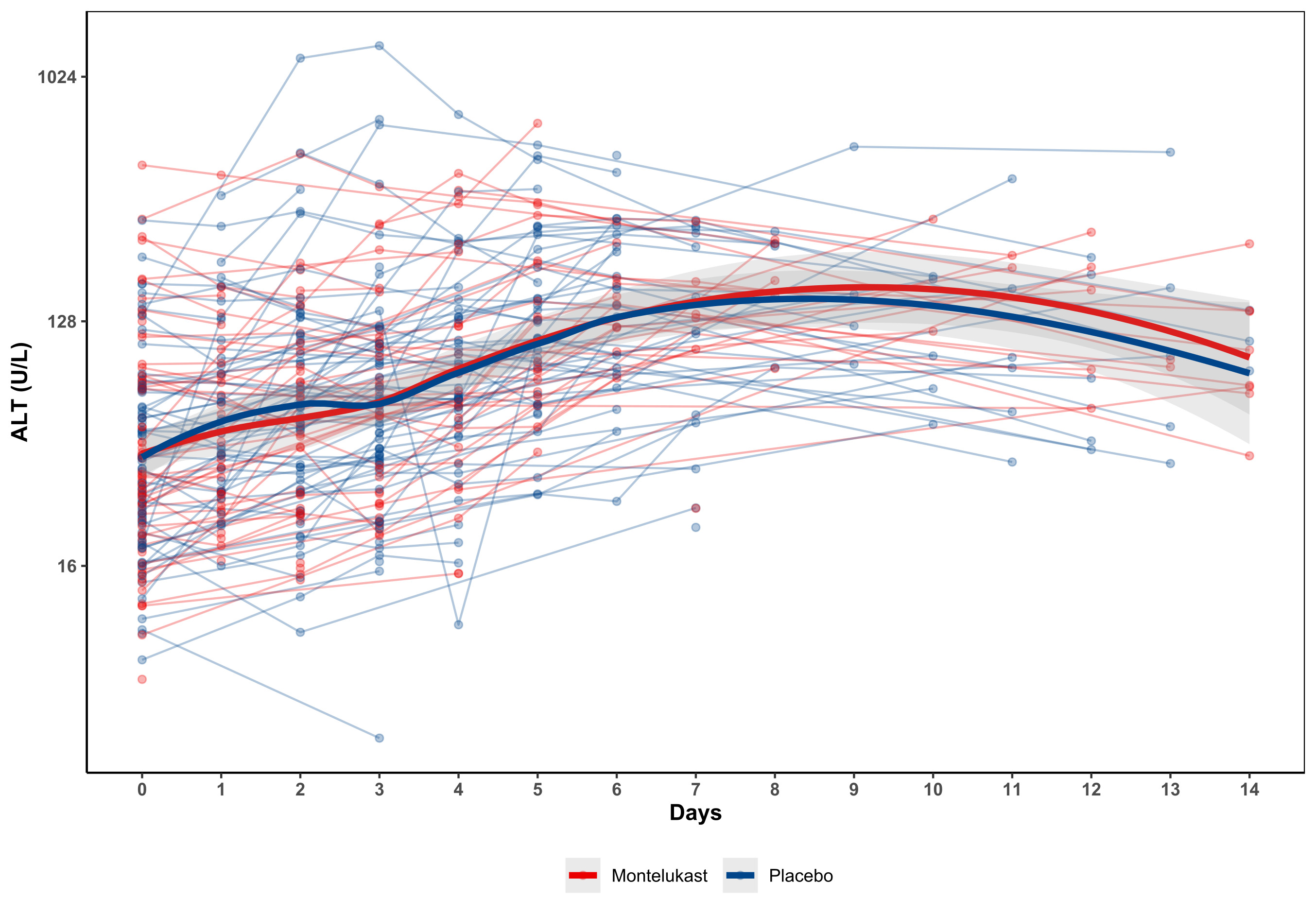

Supplement: S2 Fig — (JPG) [file pntd.0011927.s004.jpg]

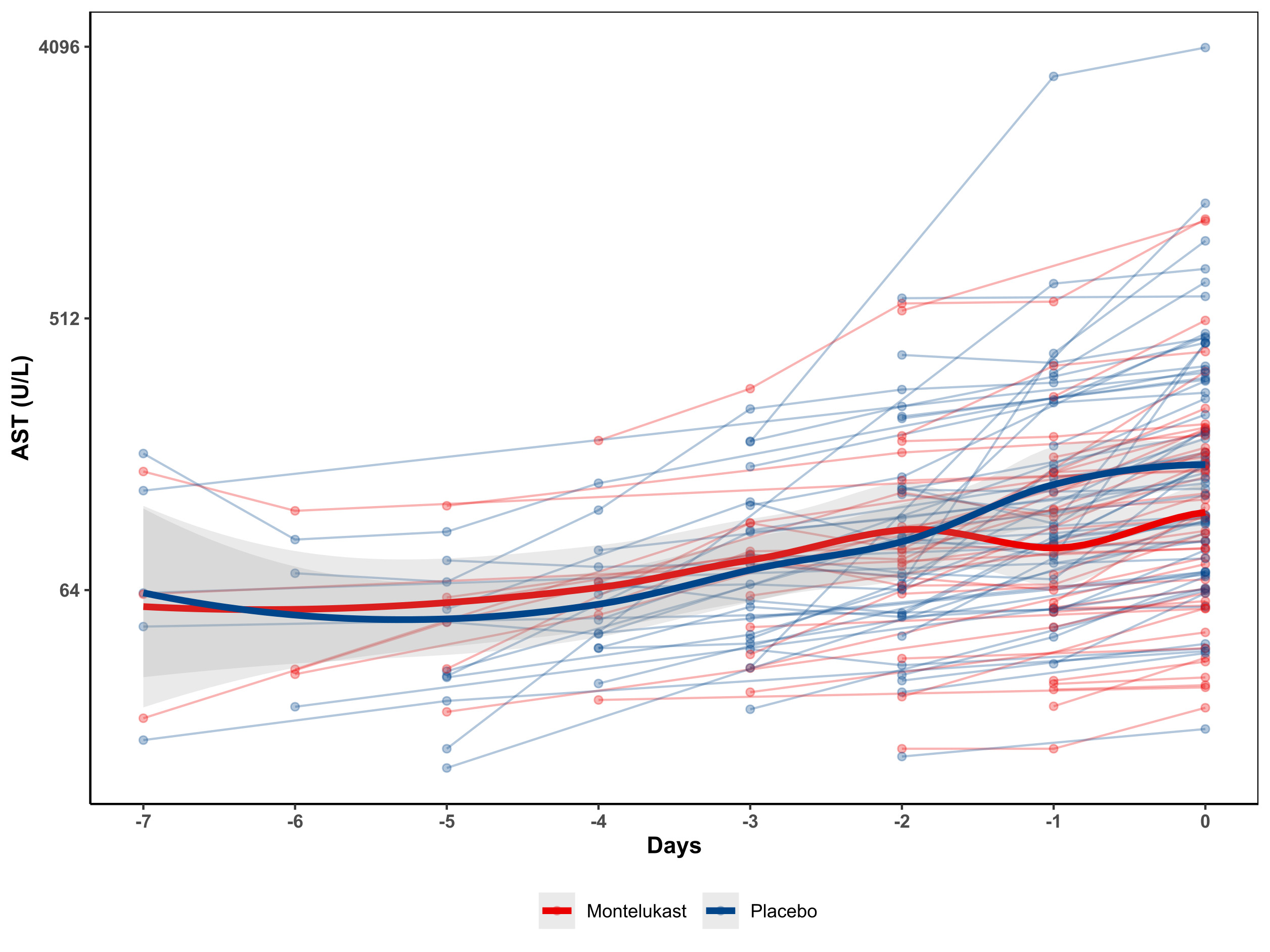

Supplement: S3 Fig — (JPG) [file pntd.0011927.s005.jpg]

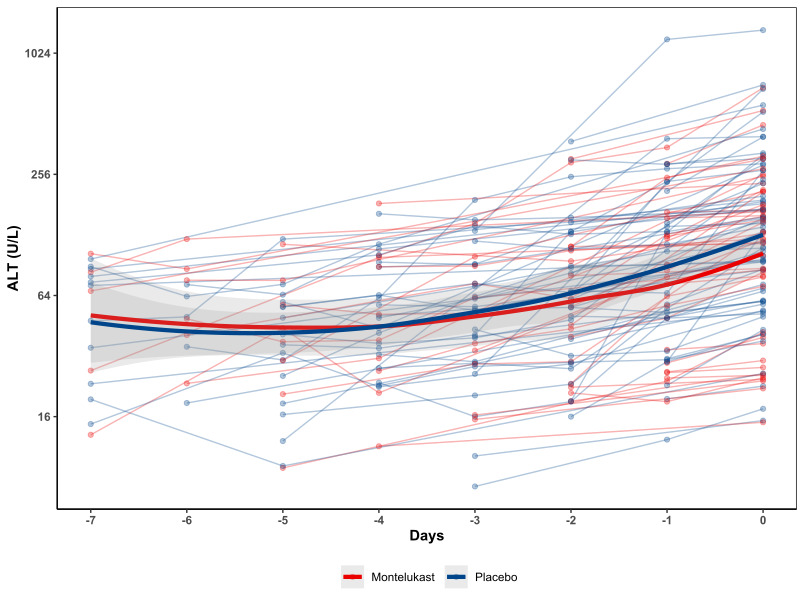

Supplement: S4 Fig — (JPG) [file pntd.0011927.s006.jpg]

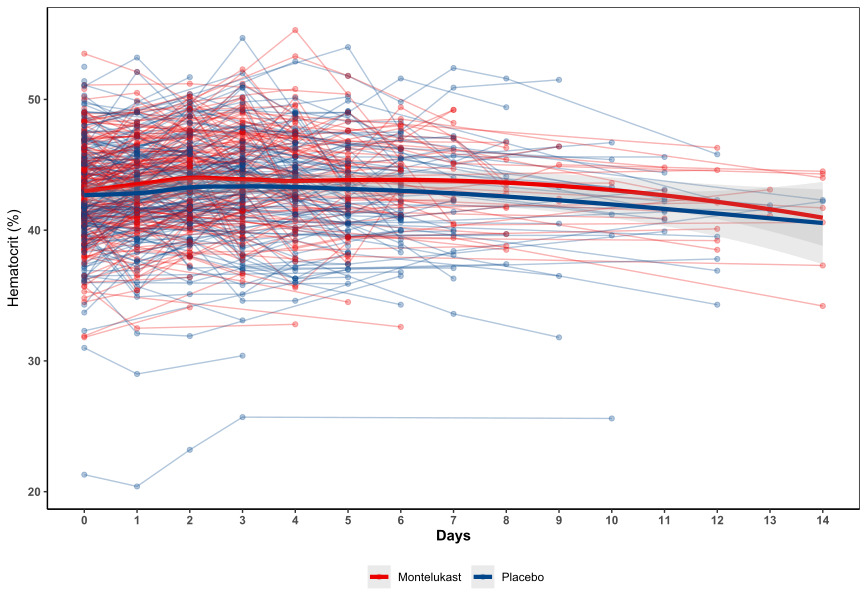

Supplement: S5 Fig — (JPG) [file pntd.0011927.s007.jpg]

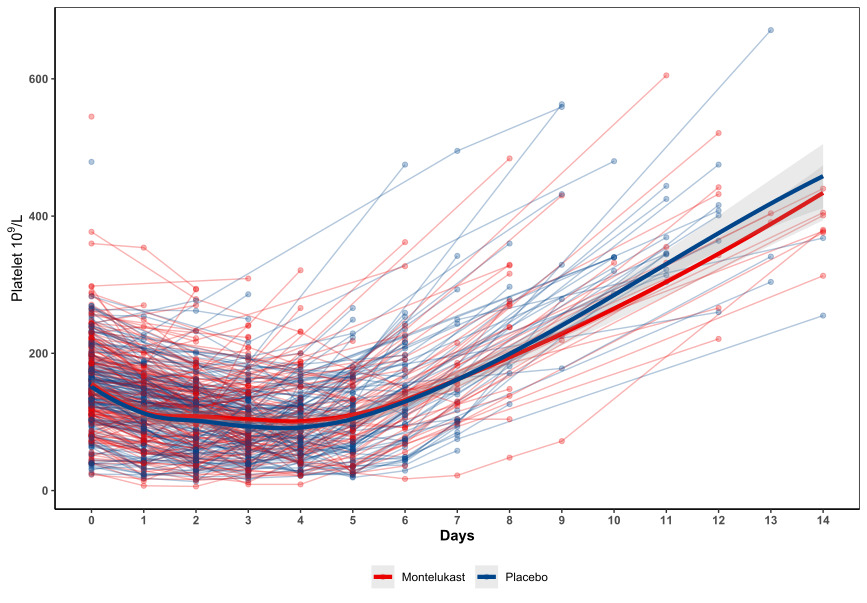

Supplement: S6 Fig — (JPG) [file pntd.0011927.s008.jpg]

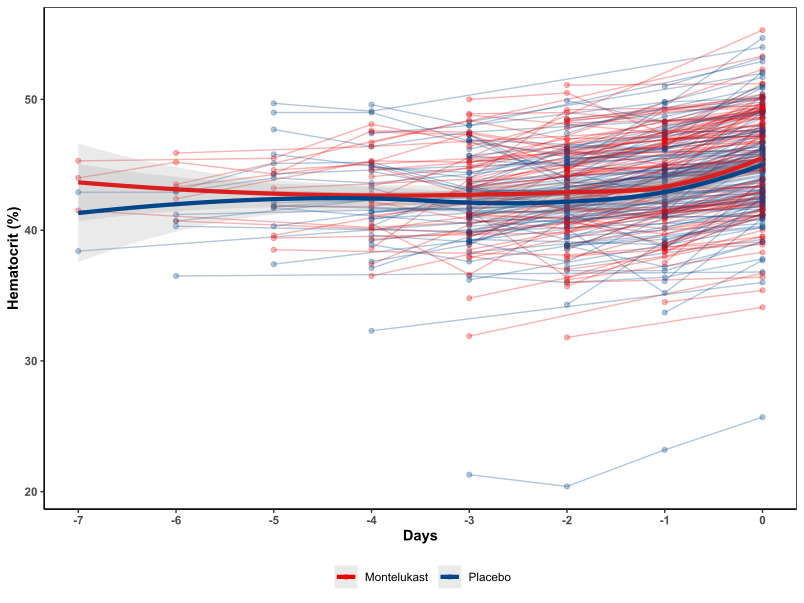

Supplement: S7 Fig — (JPG) [file pntd.0011927.s009.jpg]

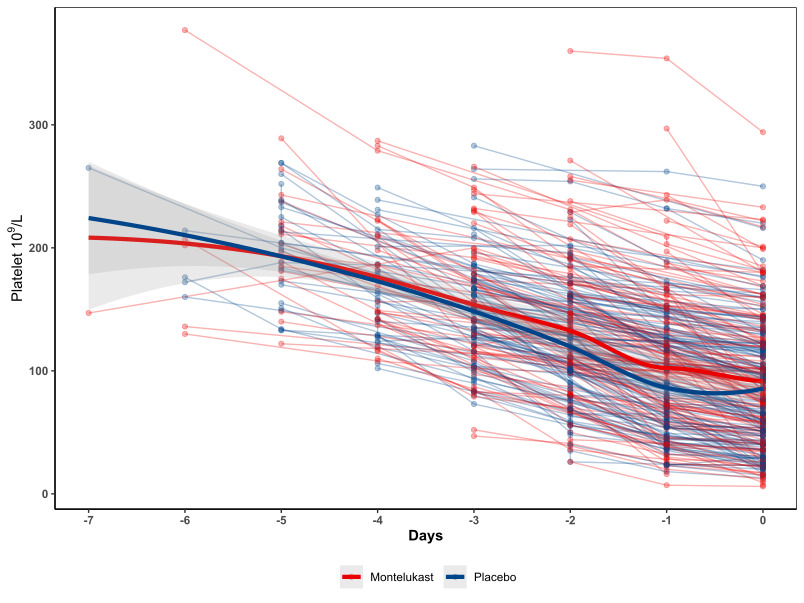

Supplement: S8 Fig — (JPG) [file pntd.0011927.s010.jpg]
