# Supplementary material for: Effect of montelukast in preventing dengue with warning signs among patients with dengue: A multicenter, randomized, double-blind, placebo-controlled trial
Source: PLoS Negl Trop Dis. 2024 Feb 2;18(2):e0011927. doi: 10.1371/journal.pntd.0011927 (PMC10866515; doi:10.1371/journal.pntd.0011927)
Supplement: S1 Table — (DOCX) [file pntd.0011927.s011.docx]

S1 Table Results of the generalized estimating equation (GEE) examining the association of various laboratory results and body temperature with the treatment groups and days until peak.

|  | Intercept | p value | Treatment  Estimate | p value | Day | p value | Treatment×  Day | p value |
| --- | --- | --- | --- | --- | --- | --- | --- | --- |
| Body temperature (°C) | 36.74 | <0.0001 | 0.16 | 0.065 | -0.36 | <0.0001 | 0.06 | 0.127 |
| Hematocrit (%) | 44.24 | <0.0001 | 0.61 | 0.140 | 0.70 | <0.0001 | 0.21 | 0.071 |
| White blood cell | 3.17 | <0.0001 | 0.16 | 0.379 | -0.84 | <0.0001 | 0.19 | 0.242 |
| Absolute Neutrophil | 1.64 | <0.0001 | 0.08 | 0.537 | -0.53 | <0.0001 | 0.07 | 0.358 |
| Absolute Lymphocyte | 0.81 | <0.0001 | 0.02 | 0.724 | -0.06 | <0.0001 | -0.05 | 0.017 |
| Platelet | 91.53 | <0.0001 | 9.59 | 0.174 | -21.88 | <0.0001 | 0.38 | 0.846 |
| ALT | 133.11 | <0.0001 | -31.04 | 0.078 | 16.08 | <0.0001 | -4.15 | 0.190 |
| AST | 213.84 | <0.0001 | -87.88 | 0.023 | 36.36 | <0.0001 | -22.93 | 0.049 |
