# Supplementary material for: Effect of montelukast in preventing dengue with warning signs among patients with dengue: A multicenter, randomized, double-blind, placebo-controlled trial
Source: PLoS Negl Trop Dis. 2024 Feb 2;18(2):e0011927. doi: 10.1371/journal.pntd.0011927 (PMC10866515; doi:10.1371/journal.pntd.0011927)
Supplement: Attachment — Submitted filename: Response.docx [file pntd.0011927.s012.docx]

*Authors’ Response to the Review Comments*

We appreciate the constructive comments from you and the reviewers, which provide such insightful guidance for the improvement of the quality of the paper. We carefully considered comments offered. Herein, we explain how we revised the paper based on those comments and recommendations.

We hope that these revisions improve the paper such that you and the reviewers now consider it worthy of publication. Please, find below the referees’ comments repeated in italics and our responses inserted after each comment. To facilitate the work of the reviewers, in some instances we refer to the former manuscript indicating the line number.

**Response to Comments from Reviewer #2:**

Comment 1:

*It is recommended to increase the font size of the axes in figure 2.*

Response:

The font size of both figure 2 and 3 has been increased as suggested.

Comment 2:

*In a personal opinion, it is not recommended to use e-mail for the traffic of sensitive information. For future trials, the use of software with encrypted data is recommended.*

Response:

Thank you for the recommendation. The concern will be taken into consideration for future trials.

Comment 3:

*The highest incidence strain in the trial was DENV-1 What would be the justification for it not being relevant at the time of the trial?*

Response:

The observed incidence of serotypes was as expected. In accordance with a previous study (Kerdpanich P, et al. Am J Trop Med Hyg. 2020 Dec 14;104(3):1058-1066.), the incidence of prevalent serotypes in this region has shown a pattern of shifting every one to two years. Therefore, the two-year duration of the study allowed for the observation of only one prevailing serotype. This issue has been added to the limitations of the study (L391).

**Response to Comments from Reviewer #3:**

Comment 1:

*Incidence rate of transaminase levels in previous 2018 open-label retrospectively study could be cited as reference in this paper.*

Response:

The study has been cited (L367) as suggested.

Comment 2:

*Data and safety monitoring board members composition that reviewed the study results could be added in the methods section.*

Response:

Thank you for the comment. The names of the board members were mentioned in the acknowledgement due to space limitation in the methods section.

Comment 3:

*Yes, safety results could be elaborated with graph/mean values showing change from admission of lab levels of hematocrit, platelets, transaminase levels after enrolment. As transaminase levels were defined as combination of AST and ALT concentrations of more than 3 times than normal, it would be better to include table or graph showing these levels change from baseline to clinical outcome.*

Response:

These graphs were included in the supplementary appendices.

Comment 4:

*Observed Transaminase levels were higher in Montleukast group, however no serious adverse event were reported. This should be presented in safety results with investigator causality assessment to study drug.* *Data safety and monitoring board recommendations following review of interim analysis could be added in the paper.*

Response:

We apologize for any confusion. The observed transaminase levels were *significantly lower* in the Montelukast group, as analyzed by GEE (Supplementary Table 1). However, baseline characteristics showed that AST was slightly higher, while ALT was slightly lower in the Montelukast group. These baseline transaminase levels were not subjected to statistical testing due to the true randomization nature.

The data safety and monitoring board conducted interim analyses, ensuring there were no concerns about the efficacy and safety of montelukast. It is important to note that, during interim analyses, the differences in transaminase levels were not significant, especially with a Lan-DeMets spending function and O’Brien–Fleming monitoring boundary.

Additionally, it is worth mentioning that transaminase elevation is not categorized under the CTCAE terms, and in this study, the observed transaminase elevations did not meet the criteria for hepatic failure as defined in CTCAE.
